# Predicting Hospital Demand During the COVID-19 Outbreak in Bogotá, Colombia

**DOI:** 10.3389/fpubh.2020.582706

**Published:** 2020-11-11

**Authors:** Claudia Rivera-Rodriguez, Beatriz Piedad Urdinola

**Affiliations:** ^1^Department of Statistics, University of Auckland, Auckland, New Zealand; ^2^Department of Statistics, National University of Colombia, Bogotá, Colombia

**Keywords:** COVID-19, SEIR, Bogotá, compartmental model, Colombia

## Abstract

Colombia, like many developing nations, does not have a strong health system able to respond to a pandemic of the magnitude of Covid-19. There is an increasing need to create a model that allows particular clinics and hospitals to estimate the number of patients that require Intensive Care Units-ICU care (critical), and the number of patients that require hospital care (severe), but not ICU care, in order to manage their limited resources. This paper presents a prediction of the total number of ICU and regular beds that will be needed in Bogotá, Colombia, during the COVID-19 pandemic. We use an SEIR model that includes three different categories of infection: those who can stay at home, those who need regular hospital beds, and those who need ICU treatment. The model allows for a time varying transmission rate which we use to incorporate the measures introduced by the government over the period of one semester. The model predicts that by mid November 2020, the city will need 1362 ICU beds and more than 9000 regular hospital beds. The number of active cases will be 67,866 by then and the death toll will reach 13,268 people by the end of December. We provide a Shiny app available at https://claudia-rivera-rodriguez.shinyapps.io/shinyappcovidclinic/. The original values in the app reproduce the results of this paper, but the parameters and starting values can be changed according to the user's needs. COVID-19 has posed too many challenges to health systems around the globe. This model is a useful tool for cities, hospitals and clinics in Colombia that need to be prepared for the excess demand of services that a pandemic like this one generates. Unfortunately, the model predicts that by mid-November the projected capacity of the system in Bogotá will not be enough. We expect the lockdown rules to be strengthened in future days, so the death toll will not be as bad as predicted by this model.

## 1. Background

The novel coronavirus disease 2019 (COVID-19) epidemic had spread from China to almost all the countries in the world by April 1, 2020. The first official case was reported in Colombia on March 6, 2020, from an imported case, and evolved to local cases of transmission. In order to reduce the impact of the COVID-19 outbreak in Bogotá, the largest city in Colombia, a local lockdown was introduced on March 15, 2020, followed by a national lockdown on March 19, 2020. Colombia, like many developing nations, does not have a strong health system able to respond to a pandemic of the magnitude of the present one. Neither in terms of infrastructure and medical personnel, nor in terms of logistical preparedness and the technical capacity to provide all medically needed resources. The latter is the main motivation to create a model that allows particular clinics and hospitals to estimate the number of beds and respirators needed during the peak days. Specifically, we are interested in estimating the number of patients that require Intensive Care Units-ICU care (critical), and the number of patients that require hospital care (severe), but not ICU care.

As of April 4, 2020, Colombia had only carried out 460 tests per million people (https://infogram.com/, https://ourworldindata.org/covid-testing), whereas other countries, such as Germany and South Korea, had carried out over 1,000 tests per million people. Additionally, on March 26, one of the two available machines used to run the detection tests broke, leading to a reduction operations and causing delays in the detection of the total number of cases. Unfortunately, for developing countries like Colombia, it has been an enormous effort to expand facilities and the production of biotechnology inputs to run the necessary number of tests required to detect all active cases of the virus; the highest number up to date has been 17,000 tests, on June 19, 2020. Thus, one of the biggest concerns is that the data may not be well-informative as to how many hospital beds (and ICU beds) will be needed during the peak of the outbreak. In fact, one of the main caveats for this study is that the official data is very likely to be under estimated, as only patients with at least one symptom or that have had contact with another detected case are being tested (1) (National Health Institute by its acronym in Spanish). Moreover, we are employing an overall probability of requiring ICU treatment, although sex, age, and co-morbidity (diabetes, hypertension, acute respiratory diseases, and depressed immune system) give rise to differential probabilities, that are not taken into account here.

We implemented an SEIR model (Susceptible - Exposed - Infectious - Recovered) to forecast the number of cases in Bogotá, the largest city in Colombia and the one with the largest numbers of cases to date, using the public official COVID-19 information from the Health Secretariat-Saludata and available at (http://saludata.saludcapital.gov.co/osb). The model includes three different categories of infection: Infected that require ICU care, Infected that require hospital care, but not ICU care, and Infected that only require Home care. The model accounts for the effect of control strategies introduced by the government by changing the transmission rate over time. We developed a Shiny app that displays the results from the model. It is publicly available at (https://claudia-rivera-rodriguez.shinyapps.io/ shinyappcovidclinic/). Users can change the initial parameters according to their specific situation. The Shiny app can work as a forecasting tool for individual clinics by specifying the market share (percentage) of the population corresponding to the clinic. During the outbreak, some clinics should be ready to see an increase in their market share because they may have more resources, such as ICU beds, and the model allows each clinic to adjust this. The model can be used for specific cities or towns: the user only needs to change the population size and some of the parameters of interest.

## 2. Methods

SIR methods (Susceptible-Infected-Recovered) have become widespread in the prediction of communicable diseases since their creation in the early 20th century (2). Several authors have provided forecasting models using this method, as presented in (3), but SIR models rely heavily on initial assumptions that are strong. SEIR models are a variation that relaxes some of those assumptions, including closed populations, and account for communicable diseases that transmit in transitions, starting from the entire population (Susceptible) that incubate the disease for a period of time (Exposed) making the person infected but not infectious (I) and finally become Recovered (*R*) (4). Each transition has a rate based on what is observed from a population, that is, a susceptible person gets infected at a transmission rate once in contact with an infected individual, and becomes exposed. Once exposed, the transition to infected happens at a rate that captures the inverse of the mean latent period of the disease. The final transition is recovery with permanent immunity. We chose this model to estimate the demand for beds for each institution in Colombia, distinguishing between regular and ICU (Intensive Care Units) beds, which allows different transition rates for each type. We also estimate the requisite preparedness and logistical needs for the health providers. Similar methods have been used to forecast similar needs in Europe and the United States of America (5–7) and more recently they have also been part of the discussion in developing nations: SIR models are also used to forecast the virus progression in Colombia.

## 3. Model

We fitted a deterministic SEIR model over 6 months. For practical purposes, it is important to bear in mind that policies were changing over this period of time, and therefore models must be updated. The population is divided into compartments or states that individuals transition from one state to the other, corresponding to Susceptible (*S*), Exposed (*E*), Infected (*I*), recovered (*R*), and death (*D*). Those Infected (*I*) are subdivided into three compartments: *I*_U_, *I*_NoU_, *I*_H_ which, respectively, denote infected individuals that require ICU care, infected individuals that require hospital care but not ICU, and infected individuals that only require home care.

**Figure 1:**
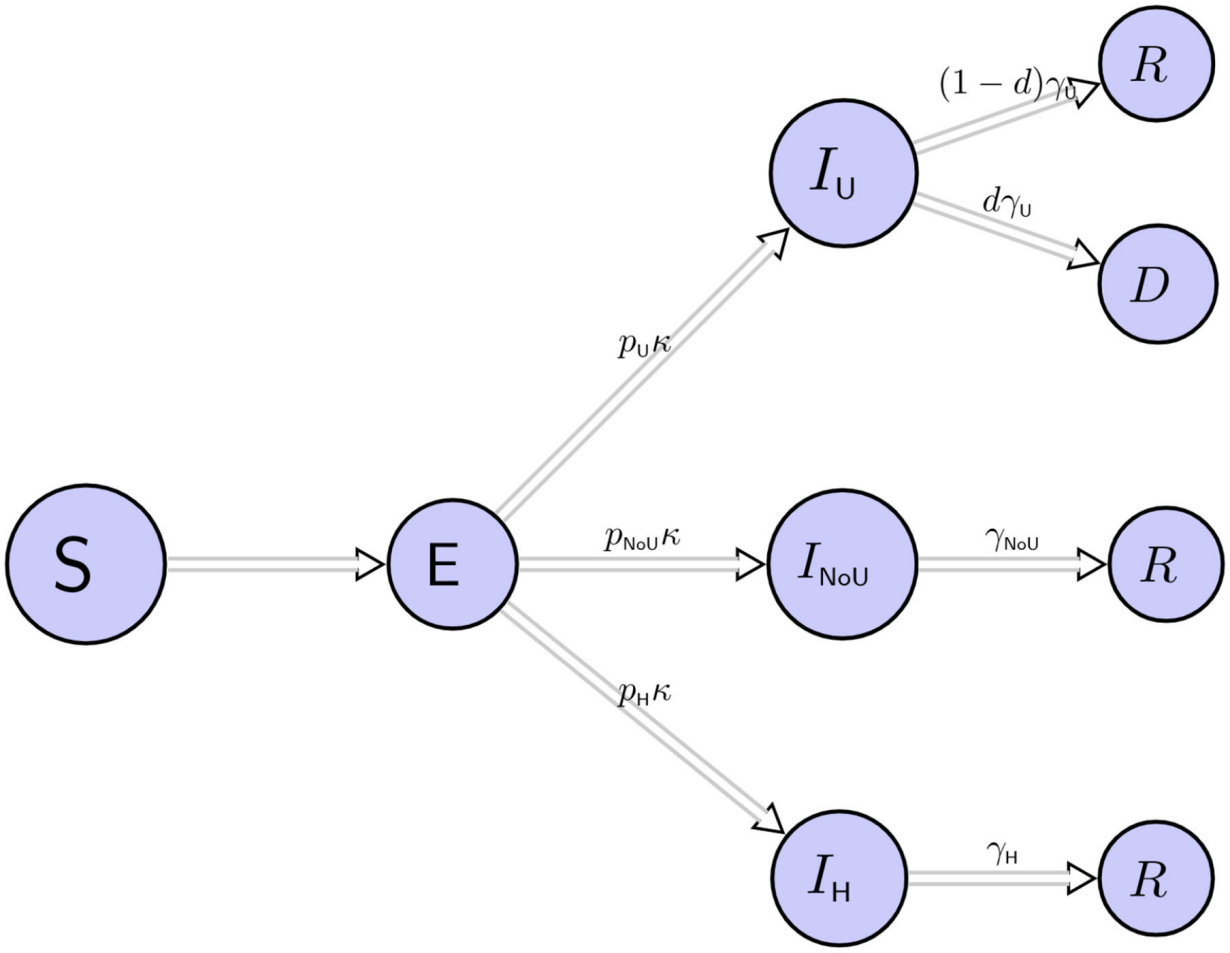
We divided the population into susceptible (*S*), exposed (*E*), infected ICU (*I*_U_), infected in hospital but not ICU (*I*_NoU_), infected that require only home care (*I*_H_), recovered (*R*), and dead subjects (*D*). Infected subjects are *I*_U_, *I*_NoU_ or *I*_H_ with probabilities *p*_U_, *p*_NoU_ and *p*_H_, respectively. The term 1/κ is the mean incubation period and γ_U_, γ_NoU_, γ_H_ are the daily probabilities that the respective patients recover. *d* is the probability of death for ICU patients.

One implication of our model is that it does not consider events such as births or migration, and it only considers deaths due to COVID-19. Note that we assume that patients transit from E to ICU care directly, therefore we assume that the average time from (E) to (*I*_U_) is larger than the average time from (E) to (*I*_NoU_) and subsequently this is larger than the average time from (E) to (*I*_H_). These transitions and considerations are summarized in Figure 1. We also assume that the only patients that transition to death are those in ICU, whilst other infected patients recover: this assumption is based on the fact that at the beginning of the pandemic, when this document was written, there was no collected evidence besides patients in clinics, and the observed rates showed a disproportionately large mortality rate for ICU patients in Wuhan and Washington State (8, 9). The total population of Bogotá is 7.4 million, but we assume an initial population size of 8 million to account for its metropolitan area because people commute daily to work and study from the surrounding towns to Bogotá's Capital District.

We describe the epidemic transitions through the model using the following equations.

(1) $$\begin{array}{l} \frac{dS\left(t\right)}{dt}=-\beta \left(t\right)S\left(t\right)\frac{\left(I_{\text{U}}\left(t\right)+I_{\text{NoU}}\left(t\right)+I_{\text{H}}\right)\left(t\right)}{N} \end{array}$$

(2) $$\begin{array}{l} \frac{dE\left(t\right)}{dt}=\beta \left(t\right)S\frac{\left(I_{\text{U}}\left(t\right)+I_{\text{NoU}}\left(t\right)+I_{\text{H}}\left(t\right)\right)}{N}\\ \;-\left(\kappa_{\text{U}}+\kappa_{\text{NoU}}+\kappa_{\text{H}}\right)E\left(t\right) \end{array}$$

(3) $$\begin{array}{l} \frac{dI_{\text{U}}\left(t\right)}{dt}=\kappa_{\text{U}}E\left(t\right)-\gamma_{\text{U}}I_{\text{U}}\left(t\right) \end{array}$$

(4) $$\begin{array}{l} \frac{dI_{\text{NoU}}\left(t\right)}{dt}=\kappa_{\text{NoU}}E\left(t\right)-\gamma_{\text{NoU}}I_{\text{NoU}}\left(t\right) \end{array}$$

(5) $$\begin{array}{l} \frac{dI_{\text{H}}\left(t\right)}{dt}=\kappa_{\text{H}}E\left(t\right)-\gamma_{\text{H}}I_{\text{H}}\left(t\right) \end{array}$$

(6) $$\begin{array}{l} \frac{dR\left(t\right)}{dt}=\left(1-d\right)\gamma_{\text{U}}I_{\text{U}}\left(t\right)+\gamma_{\text{NoU}}I_{\text{NoU}}\left(t\right)+\gamma_{\text{H}}I_{\text{H}}\left(t\right) \end{array}$$

(7) $$\begin{array}{l} \frac{dD\left(t\right)}{dt}=d\gamma_{\text{U}}I_{\text{U}}\left(t\right)+\gamma_{\text{NoU}}I_{\text{NoU}}\left(t\right)+\gamma_{\text{H}}I_{\text{H}}\left(t\right) \end{array}$$

(8) $$\begin{array}{l} N=S\left(t\right)+I\left(t\right)+R\left(t\right)+D\left(t\right) \end{array}$$

where κ_NoU_ = *p*_NoU_κ, κ_U_ = *p*_U_κ and κ_H_ = *p*_H_κ. This set of equations is the core model, where, at time *t*, the population is divided into susceptible (*S*(*t*)), exposed (*E*(*t*)), infected ICU (*I*_U_(*t*)), infected in hospital but not ICU (*I*_NoU_(*t*)), infected that require only home care (*I*_H_(*t*)), recovered (*R*(*t*)) and dead subjects (*D*(*t*)) individuals. The total of infected individual is *I*(*t*) = *I*_U_(*t*) + *I*_NoU_(*t*) + *I*_H_(*t*), with respective proportions *p*_U_, *p*_NoU_, and *p*_H_. The transmission rate, β(*t*), controls the rate of spread, i.e., the probability of transmitting disease between a susceptible and an infectious individual. We allow this to be a step-wise function to adjust for the measures taken by local authorities to control the spread of the virus. The term 1/κ represents the mean incubation period and γ_U_, γ_NoU_, γ_H_ are the daily probabilities that the respective patients recover. Furthermore, *d* denotes the probability of death for ICU patients. The model's transitions are described in Figure 1. Equation (1) describes the rate at which new individuals are exposed, this rate is $\beta \left(t\right)S\left(t\right)\frac{I\left(t\right)}{N}$; Equation (2) describes the rate at which exposed individuals become infected, this rate is (κ_U_ + κ_NoU_ + κ_H_)*E*(*t*). Infected individuals become infected in one of the three categories: *I*_U_(*t*) + *I*_NoU_(*t*), and *I*_H_(*t*) with probabilities *p*_U_, *p*_NoU_, and *p*_H_, respectively. Additionally, Equation (3) describes the rate at which infected individuals in *ICU* either die or recover, this rate is γ_U_*I*_U_(*t*). Similarly, Equation (4) represents the rate at which infected individuals in hospital (but, not *ICU*) either dies or recover, this rate is γ_NoU_*I*_NoU_(*t*) and Equation (5) describes the rate at which infected individuals at home either die or recover: γ_H_*I*_H_(*t*). Equation (6) describes the rate at which infected individuals recover. Note that this rate is always positive and the number of recovered individuals never decreases. Similarly, Equation (7) describes the rate at which infected individuals die. Note that only individuals from *ICU* die and this rate is also positive. The total population is *N* = *S*(*t*) + *I*(*t*) + *R*(*t*) + *D*(*t*) (Equation 8).

We are aware that other variables, beyond total population counts, such as age and sex distribution and having an identified co-morbidity such as obesity, diabetes, hypertension, and/or cancer, increases the probability of developing complications due to Covid-19 that increase the chances of dying. However, Colombian data was not available at the microdata level when the pandemic erupted, and still is not available, not even in tabular form, including any of these additional variables. Hence, the best we could do was to implement the model for the general observed numbers.

To model the impact of the interventions introduced by the government, we allow the transmission rate to be a step-wise function β(*t*), with three steps at *t*_0_, *t*_1_ and *t*_2_. The time *t*_0_ (2020-05-24) corresponds to the time when we start predicting, *t*_1_ (2020-06-16) is when new measures were introduced, and *t*_2_ (2020-06-30) is the date when measures were revised and implemented. We estimate β(*t*_0_) from the basic reproduction number such that *R*_0_ = 1.1 (10). For *t* > *t*_0_, we choose β(*t*) such that *R*(*t*) ≈ 1.3, for *t*_1_ ≤ *t* < *t*_2_ and *R*(*t*) ≈ 1.2, for *t* ≥ *t*_2_ (10) (Appendix).

The terms *p*_U_, *p*_NoU_ and *p*_H_ denote the probabilities that a case requires ICU care, hospital non-ICU care, and only home care, respectively. Note that *p*_U_ + *p*_NoU_ + *p*_H_ = 1. To estimate these probabilities, we use information from the Colombian National Health Institute, finding *p*_U_ = 0.0168, *p*_NoU_ = 0.14 and *p*_H_ = 0.843. The parameter κ is the daily probability of an exposed individual becoming infected, and γ_U_, γ_NoU_, γ_H_ are the daily probabilities that an infected individual recovers given that they are in ICU, Regular bed and home, respectively. The probability *d* denotes the probability that an infected ICU individual dies. Table 1 displays the parameters of the models, their interpretation and sources. The starting values for the model are based on the numbers from Bogotá, Colombia reported by May 24. There where 7,166 cases, 1,318 recovered, and 212 deaths by then in the city.

**Table 1:** Parameters and definition of model (1).

**Symbol**	**Definition**	**Value**	**Source**
β(*t*)	Transmission rate	Stepwise function	(11, 12)
κ	Daily probability of an exposed individual becoming infected: κ = 1/α, with α being the mean incubation period	1/5.2	(13)
*p*_U_	Probability of patient being ICU	0.0168	(10)
*p*_NoU_	Probability of patient being in Hospital, but not ICU	0.14	(10)
*p*_H_	Probability of patient being mild/at home	0.843	(10)
γ_U_	Daily probability that an infected individual in ICU recovers, when the mean infection period is *b*_U_, γ_U_ = 1/*b*_U_	1/6	(13–15)
γ_NoU_	Daily probability that an infected individual in Hospital, but not ICU, recovers, when the mean infection period is *b*_NoU_, γ_NoU_ = 1/*b*_NoU_	1/5	(13–16)
γ_H_	Daily probability that an infected individual in Hospital, but not ICU, recovers, when the mean infection period is *b*_H_, γ_H_ = 1/*b*_H_	1/5	(13–16)
*d*	Probability of dying given that patient is in ICU	0.50	(17)

## 4. Results

**Figure 2:**
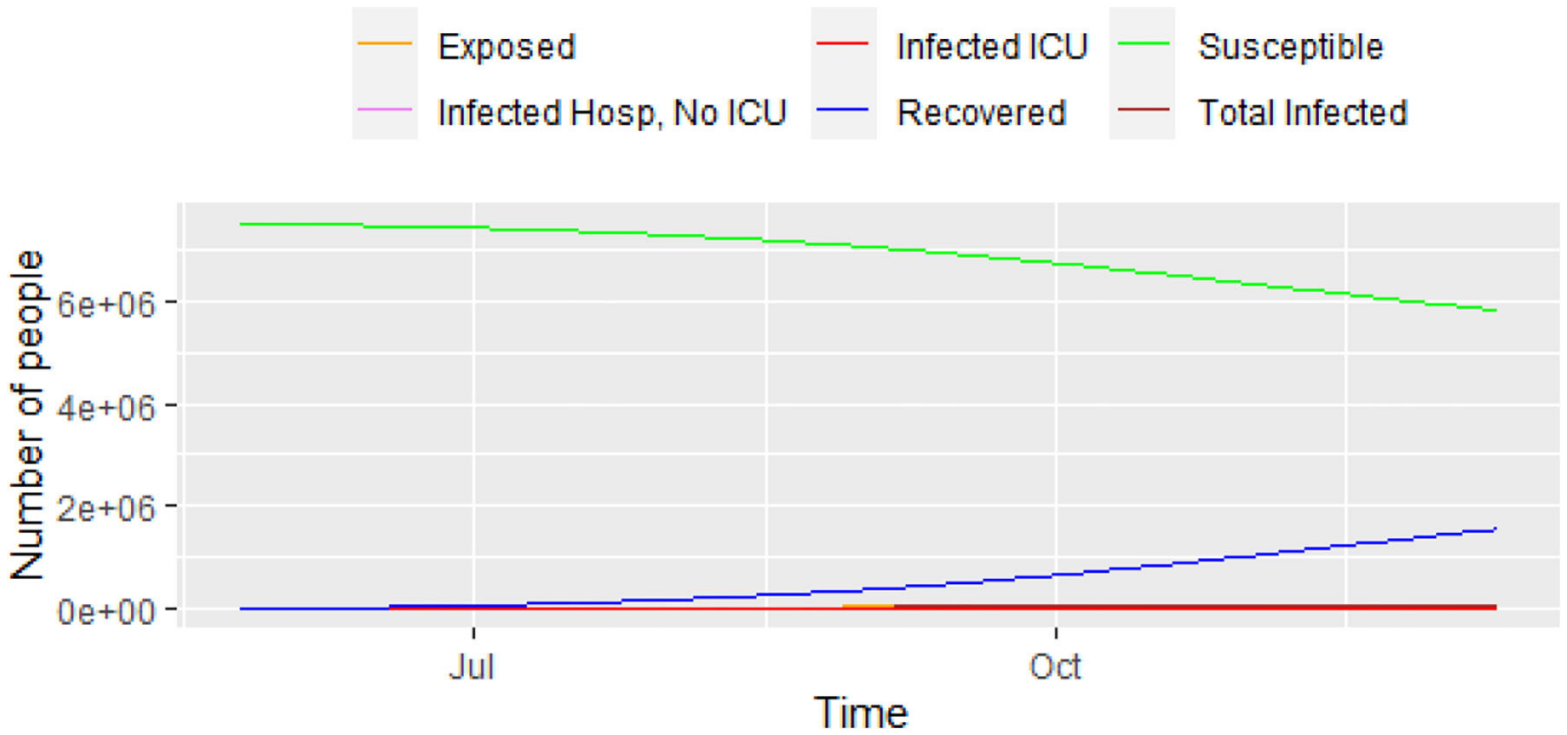
Progress of the epidemic using model (1).

Figure 2 shows the results the model predicts for each category. Even with all the positive measures assumed in the model, we predict that the peak of the epidemic could happen around November 11, 2020. During the peak of the epidemic, the model predicts that 1,362 ICUs will be needed for coronavirus patients, and 9,470 non-ICU hospital beds. We predict that the maximum number of prevalent cases will be 67,866 (2020-11-14) for the 6 months of the prediction. With the parameters in the models, the total number of deaths could reach 13,268 in 6 months' time.

**Figure 3:**
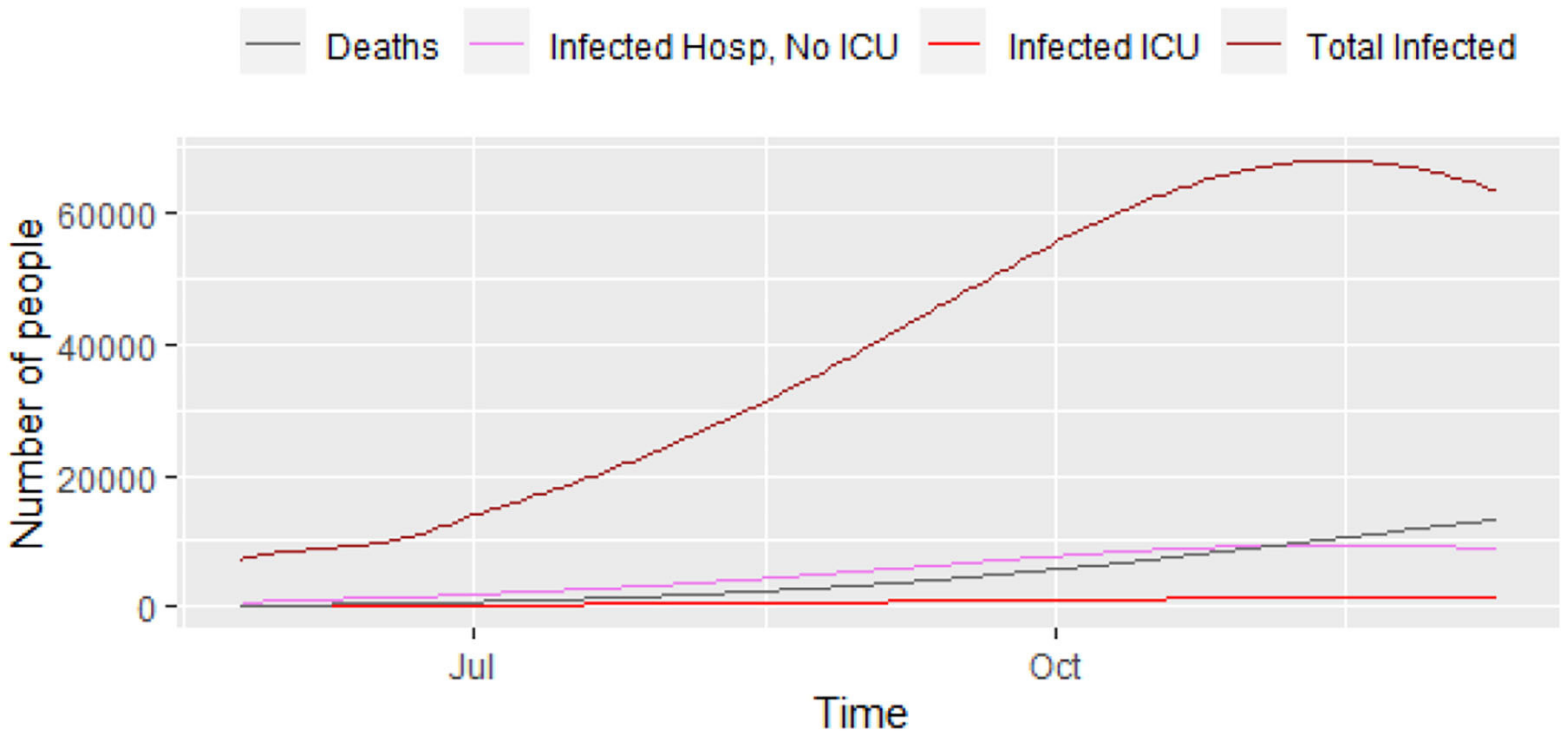
Progress of the epidemic—infected and deaths, results from model (1).

**Figure 4:**
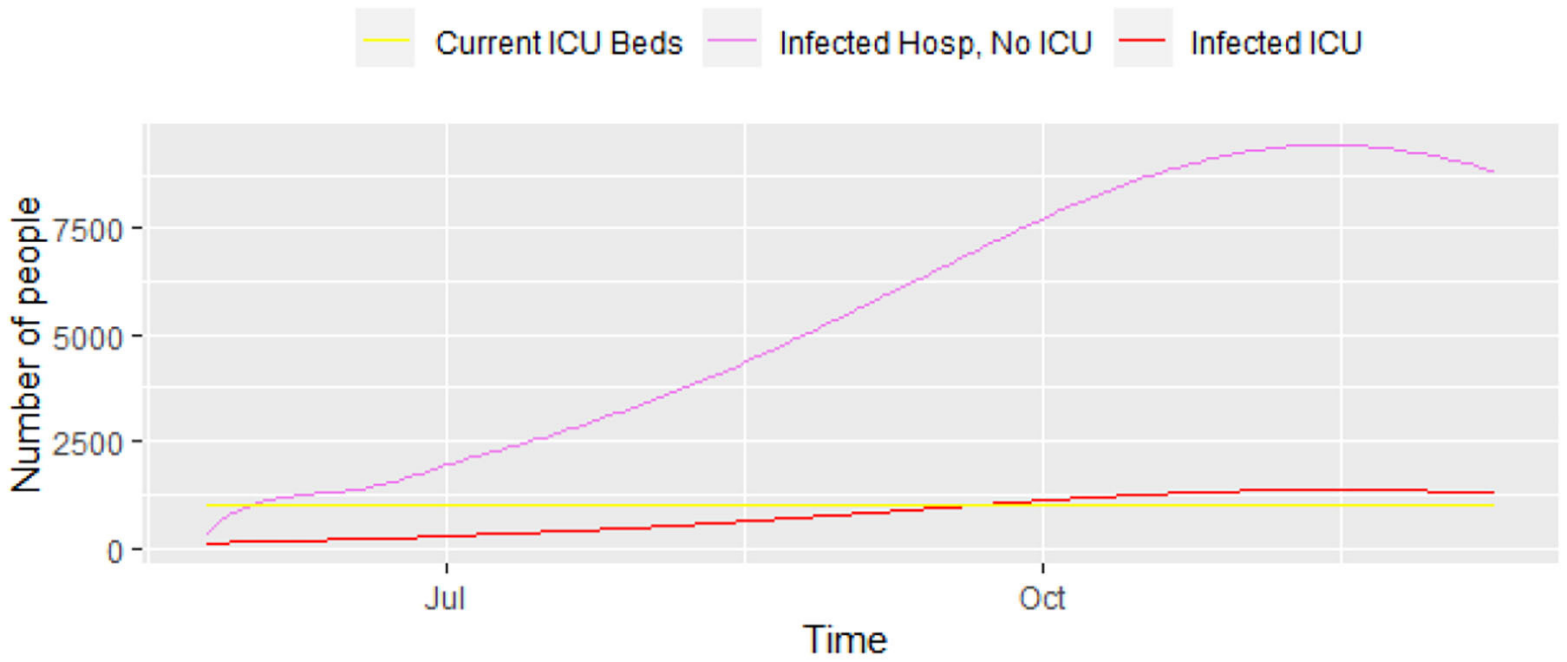
Progress of the epidemic—infected that require ICU and infected that require regular hospital beds, results from model (1).

Figure 3 displays a closer picture of those infected and the total number of deaths. We can see that the total number of infected that will need hospital care (ICU and non-ICU) is high enough for concern. Additionally, Figure 4 shows those infected that will need hospital care, compared to the current number of ICU beds in the city. It shows that the number of ICUs needed will be 1,362, i.e., the city has to increase its capacity in order to provide care to everyone that needs it. The local authorities in Bogotá are planning to have a total of 1,200 ICU beds in the city, but the current number is still lower than that. The number 1,200 will be overrun by mid-September, 2020, with a death toll of 4,850 people by then. Unfortunately, the trend keeps on increasing over the following months, which reflects the lack of preparedness for a catastrophe like the current one in Bogotá, and probably in other similar developing nations.

When we increase the number of days that ICU patients take to recover, i.e., 1/γ_U_ = 14 days, rather than 7, the number of ICU beds needed almost doubles. If the probability of being an ICU patient *p*_U_ is reduced, the number of beds is reduced, but even a small increase in this probability will cause a large increase in the number of ICU beds needed during the peak of the epidemic.

## 5. Discussion

This paper presents a prediction of the total number of ICU and regular beds that will be needed during the pandemic COVID-19 for Bogotá, Colombia. We use a SEIR model that differentiates between three types of infected patients: those who can stay at home, those who need regular hospital beds, and those who need ICU treatment. It employs a mean incubation period of 5.2 days and mean infection periods of 4 days (for patients at home), 5 days (for patients in regular hospital beds), and 7 days (for patients in ICU). The parameters assumed in the model are for a positive scenario, where the effective reproduction number during the lock-down is assumed to be 1.1, and 1.3 after the lock-down, and 1.2 when other measures are introduced. We assume that 2.6% of patients require ICU treatment, 13.4% require regular hospital beds, and the rest only require home care. The model allows for a time varying transmission rate which we use to incorporate the measures introduced by the government over the period of 1 year. The model predicts that by mid-November, 2020, the city will reach the peak of the epidemic with a total 67, 866 prevalent cases and 1,362 active ICU prevalent cases.

The number of patients that need hospitalization can surpass the current planned capacity, set at 1,200 beds for ICU beds in the city, and the death toll can reach a total of 13,268 in 6 months' time (by the end of December). The unpreparedness of the health system will only increase COVID-19 related and unrelated mortality, as already observed in Italy, the USA, and other countries. Measures like lockdown have been used in most countries to diffuse the demand for health services due to COVID-19 over time, however it may be insufficient if there are not enough resources to ramp the health services in developing nations, such as is the case of Colombia, where the need for additional resources is a priority at this point.

Other than the intrinsic limitations of SEIR models, this prediction model does not take into account the age and sex distribution of the population, but we plan to introduce such distinctions in a future version of the model with an additional mixing including the contact matrices, as the recent national population census in Colombia is available. Also, we have fitted a model with two interventions: a lockdown and mitigation measures, but this can be modified later in time. Neither do we take into account regional differences, in a tropical context relate to weather and climate, because there is no evidence, to date, whether the pattern of spread of the novel coronavirus depends on weatherconditions.

Finally, we provide a Shiny app available at https://claudia-rivera-rodriguez.shinyapps.io/shinyappcovidclinic/. The original values in the app reproduce the results of this paper, but the parameters and starting values can be changed according to the user's needs.

## 6. Conclusions

COVID-19 has posed too many challenges to health systems around the globe. It is remarkable that governments everywhere have swiftly responded by increasing laboratory tests, medical personnel, infrastructure, and data production linked to the disease. It is even striking that a developing nation, such as Colombia, has publicly available information updated daily on the evolution of the pandemic, with all the attendant pros and cons. Daily data is probably defective, and by now is preliminary, but still very helpful when trying to find solutions to the hard issues imposed on the demand for health resources due to the pandemic. The standard time of production of mortality data is 2 years and a trimester, according to the official national statistical office (DANE).

This model is a useful tool for cities, hospitals and clinics in Colombia that need to prepare for the excess demand of services that a situation like this imposes. The model predicts that by mid-November, the current capacity of ICUs in Bogotá will not be enough if no other measures are taken. Lock-down rules in fact were strengthened, tracking, surveillance and testing capacities also increased, and social behavior tilted toward following preventive measures. As a result, the observed reproductive numbers dramatically diminished, and when used in the model we obtain a fairly similar number of beds demanded as those actually observed, and a slightly higher mortality than observed. We expect all those measures and preventive behavior will be maintained for the remainder of the pandemic, otherwise there will be a demand for beds that will surpass the current capacity in the city.

## Data Availability Statement

The materials(code) used and/or analyzed during the current study are available from the corresponding author on reasonable request. Additionally, a shiny app is available online at https://claudia-rivera-rodriguez.shinyapps.io/shinyappcovidclinic/.

## Author Contributions

CR-R contributed to the analysis and coding of the model and the shiny app. BU contributed to model interpretation and article writing. All authors have read and approved the manuscript.

## Conflict of Interest

The authors declare that the research was conducted in the absence of any commercial or financial relationships that could be construed as a potential conflict of interest.

## Appendix

### Reproduction Number and β(*t*)

We use the next generation matrix approach to find the basic reproduction number (19, 20). We estimate β(*t*_0_) from the basic reproduction number. To find *R*_0_, following (4), we let **x** = [*E, I*_U_, *I*_NoU_, *I*_*H*_] and **y** = [*S, R, D*]. Disease free equilibrium is **x**_0_ = [0, 0, 0, 0] and **y**_0_ = [*N*, 0, 0]. Then,

(A1) $$\begin{array}{l} F_{i,t}\left(\text{x},\text{y}\right)={\left[-\beta \left(t_{0}\right)S\frac{\left(I_{\text{U}}+I_{\text{NoU}}+I_{\text{H}}\right)}{N},0,0,0\right]}^{T} \end{array}$$

(A2) $$\begin{array}{l} V_{i,t}\left(x,y\right)=[\left(\kappa_{\text{U}}+\kappa_{\text{NoU}}+\kappa_{\text{H}}\right)E,\gamma_{\text{U}}I_{\text{U}}-\kappa_{\text{U}}E,\gamma_{\text{NoU}}I_{\text{NoU}}\\ \;-{\kappa_{\text{NoU}}E,\gamma_{\text{H}}I_{\text{H}}-\kappa_{\text{H}}E]}^{T} \end{array}$$

and

(A3) $$F=\frac{F_{i,t}\left(x_{0},y_{0}\right)}{dx}=\left[\begin{array}{cccc} 0 & \beta \left(t_{0}\right) & \beta \left(t_{0}\right) & \beta \left(t\right)\\ 0 & 0 & 0 & 0\\ 0 & 0 & 0 & 0\\ 0 & 0 & 0 & 0 \end{array}\right]$$

(A4) $$V=\frac{V_{i}\left(x_{0},y_{0}\right)}{dx}=\left[\begin{array}{cccc} \left(\kappa_{\text{U}}+\kappa_{\text{NoU}}+\kappa_{\text{H}}\right) & 0 & 0 & 0\\ \kappa_{\text{U}} & \gamma_{\text{U}} & 0 & 0\\ \kappa_{\text{NoU}} & 0 & \gamma_{\text{NoU}} & 0\\ \kappa_{\text{H}} & 0 & 0 & \gamma_{\text{H}} \end{array}\right]$$

The inverse of **V** is given by

(A5) $$V^{-1}=\left[\begin{array}{cccc} \frac{1}{\kappa_{\text{U}}+\kappa_{\text{NoU}}+\kappa_{\text{H}}} & 0 & 0 & 0\\ -\frac{\kappa_{\text{U}}}{\gamma_{\text{U}}\left(\kappa_{\text{U}}+\kappa_{\text{NoU}}+\kappa_{\text{H}}\right)} & \frac{1}{\gamma_{\text{U}}} & 0 & 0\\ -\frac{\kappa_{\text{NoU}}}{\gamma_{\text{NoU}}\left(\kappa_{\text{U}}+\kappa_{\text{NoU}}+\kappa_{\text{H}}\right)} & \; & \frac{1}{\gamma_{\text{NoU}}} & 0\\ -\frac{\kappa_{\text{H}}}{\gamma_{\text{H}}\left(\kappa_{\text{U}}+\kappa_{\text{NoU}}+\kappa_{\text{H}}\right)} & 0 & 0 & \frac{1}{\gamma_{\text{H}}} \end{array}\right]$$

thus

(A6) $$\begin{array}{l} FV^{-1}\\ =\beta \left(t_{0}\right)\left[\begin{array}{cccc} -\frac{\gamma_{\text{NoU}}\gamma_{\text{H}}\kappa_{\text{U}}+\gamma_{\text{U}}\gamma_{\text{H}}\kappa_{\text{NoU}}+\gamma_{\text{U}}\gamma_{\text{NoU}}\kappa_{\text{H}}}{\gamma_{\text{U}}\gamma_{\text{NoU}}\gamma_{\text{H}}\left(\kappa_{\text{U}}+\kappa_{\text{NoU}}+\kappa_{\text{H}}\right)} & 1/\gamma_{\text{U}} & 1/\gamma_{\text{NoU}} & 1/\gamma_{\text{H}}\\ 0 & 0 & 0 & 0\\ 0 & 0 & 0 & 0\\ 0 & 0 & 0 & 0 \end{array}\right] \end{array}$$

The spectral radius of **FV**^−1^ is

(A7) $$\begin{array}{l} \rho \left(FV^{-1}\right)=R_{0}=\beta \left(t_{0}\right)\left|\frac{-\gamma_{\text{NoU}}\gamma_{\text{H}}\kappa_{\text{U}}-\gamma_{\text{U}}\gamma_{\text{H}}\kappa_{\text{NoU}}-\gamma_{\text{U}}\gamma_{\text{NoU}}\kappa_{\text{H}}}{\gamma_{\text{U}}\gamma_{\text{NoU}}\gamma_{\text{H}}\left(\kappa_{\text{U}}+\kappa_{\text{NoU}}+\kappa_{\text{H}}\right)}\right|\\ \;=\beta \left(t_{0}\right)\tau \end{array}$$

Using this, we find that

(A8) $$\beta \left(t_{0}\right)=\left|\frac{\gamma_{\text{U}}\gamma_{\text{NoU}}\gamma_{\text{H}}\left(\kappa_{\text{U}}+\kappa_{\text{NoU}}+\kappa_{\text{H}}\right)}{-\gamma_{\text{NoU}}\gamma_{\text{H}}\kappa_{\text{U}}-\gamma_{\text{U}}\gamma_{\text{H}}\kappa_{\text{NoU}}-\gamma_{\text{U}}\gamma_{\text{NoU}}\kappa_{\text{H}}}\right|R_{0}$$

When *R*_0_ = 1.1, β(*t*_0_) = 0.22. Our rationale for choosing β(*t*_1_) and β(*t*_2_) is as follows.

Using the initial β_0_, we calculate *S*(*t*_1_)/*N*, and we assume that *R*(*t*) = 1.3 for *t*_1_ ≤ *t* ≤ *t*_2_, and *R*(*t*) = 1.1 for *t* > *t*_2_.

To find β(*t*), we assume that *R*(*t*) ≈ β(*t*) * τ*S*(*t*)/*N*. So, we have that β(*t*) ≈ *R*(*t*) * *N*/(τ * *S*(*t*)) So, we have β(*t*) = 0.26 for *t*_1_ ≤ *t* ≤ *t*_2_, and β(*t*) = 0.24 for *t* > *t*_2_.

## Abbreviations

SEIR: Susceptible-Exposed-Infected-Recovered
ICU: Intensive Care Unit
COVID-19: Coronavirus disease 2019.
